# Crystallographic characterization of steel microstructure using neutron diffraction

**DOI:** 10.1080/14686996.2019.1699389

**Published:** 2019-12-02

**Authors:** Yo Tomota

**Affiliations:** a Research Center for Structural Materials, National Institute for Materials Science (NIMS), Tsukuba, Japan; b Research Institute for Measurements and Analytical Instrumentation, National Institute of advanced Industrial Science and Technology (AIST), Tsukuba, Japan; c Innovative Structural Materials Association (ISMA), Tokyo, Japan

**Keywords:** Neutron diffraction, X-ray diffraction, electron back scatter diffraction, transmission Bragg﻿-edge measurements: dilatometry, steel, phase transformation, *in situ* measurement, austenite volume fraction, 106 Metallic materials

## Abstract

Applications of neutron diffraction to microstructure evaluation of steel investigated by a project commissioned by the Innovative Structural Materials Association are summarized. The volume fraction of austenite (γ) for a 1.5Mn-1.5Si-0.2C steel was measured by various techniques including backscatter electron diffraction (EBSD) and X-ray diffraction. It is recommended to measure volume fraction and texture simultaneously using neutron diffraction. The γ reverse transformation was *in situ* monitored using dilatometry, EBSD, X-ray diffraction and neutron diffraction. The γ reversion kinetics showed excellent agreements between dilatometry and neutron diffraction, whereas the γ formation started at higher temperatures in EBSD and X-ray diffraction measurements. Such discrepancy is attributed to the change in chemical compositions at the specimen surface by heating; Mn and C concentrations were decreased with heating. Phase transformations from γ upon cooling were monitored, which enabled us to elucidate the changes in lattice parameters of ferrite (α) and γ affected by not only thermal contraction but also transformation strains, thermal misfit strains and carbon enrichment in γ in the above hypoeutectoid steel. Pearlitic transformation started after the carbon enrichment reached approximately 0.76 mass% and contributed to diffraction line broadening. Martensitic transformation with or without ausforming at 700°C was monitored for a medium carbon low alloyed steel. Dislocation density after ausforming was determined using the convolutional multiple whole profile fitting method for 10 s time-sliced data. The changes in γ and martensite lattice parameters upon quenching were tracked and new insights on internal stresses and the axial ratio of martensite were obtained.

## GRAPHIAL ABSTRACT

**Figure:**
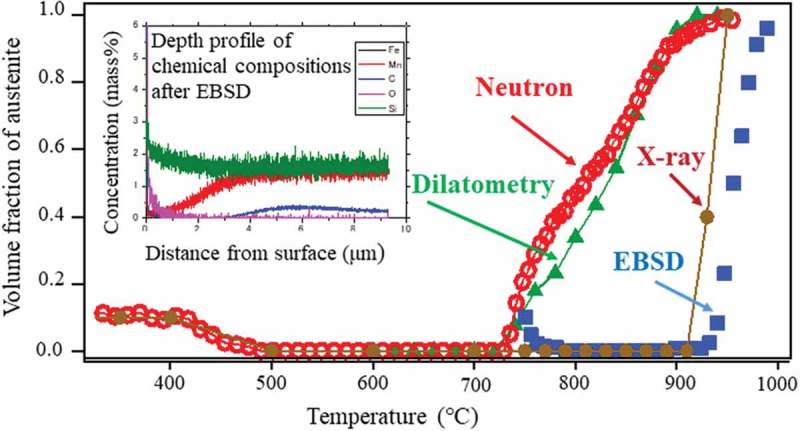

## 1. Introduction

Industrial applications of neutron scattering and diffraction measurements have recently been progressed with achieving high-intensity neutron beam at a spallation neutron source like Materials and Life Science Experiment Facility (MLF) at Japan Proton Accelerator Research Complex (J-PARC) [1] and developing complementary use of compact neutron sources [2,3]. Novel techniques using neutron diffraction, small-angle neutron scattering and transmission Bragg-edge neutron imaging have recently been developed quantitatively to evaluate microstructure in steel, the control of which is a key issue to develop advanced steels with a good balance of strength and ductility/toughness. This review is focused on neutron diffraction emphasizing how it is becoming useful to obtain global microstructural information *in situ* during material processing, which have been obtained through a project commissioned by the Innovative Structural Materials Association (ISMA)/New Energy and Industrial Technology Development Organization (NEDO) [4]. More details can be found in references [5–9].

The development of advanced steels requires quantitative measurement of microstructure, in particular, the characterization of austenite (γ) phase which plays an important role for balancing strength and ductility in low alloyed transformation-induced plasticity (TRIP) steels [10–13], quenching and partitioning (Q&P) steels [14,15], nanobainite steels [16–19] and medium Mn steels [20,21]. It is important to determine the volume fraction of γ (*fγ*) and hence several methods have been employed so far. For example, a round robin test was performed at 13 research institutes and universities in Europa on the determination of *fγ* of TRIP steel, where optical microscopy, scanning electron microscopy, X-ray diffraction, magnetic measurement, thermal diffusivity and laser ultrasonic inspection were employed [22]. As results, it was concluded that no method could be recommended because of the scattered results even using the same method. The γ in advanced steels is metastable in general, so that the method to measure the surface layer like electron backscatter diffraction (EBSD) or X-ray diffraction often results in smaller *fγ* in comparison with the result by neutron diffraction that evaluates the interior or global average value [23,24]. This is because martensitic transformation is easy to occur near the free surface. Another difficulty of *fγ* measurement is to avoid the influence of texture which has evolved during the steel production process. In case of weak texture, the following correction has frequently been adopted [25,26].

(1) $$f_{\gamma }=\frac{\frac{1}{n}\sum_{1}^{n}\frac{I_{hkl}^{\gamma }}{R_{hkl}^{\gamma }}}{\left(\frac{1}{m}\sum_{1}^{m}\frac{I_{hkl}^{\alpha }}{R_{hkl}^{\alpha }}+\frac{1}{n}\sum_{1}^{n}\frac{I_{hkl}^{\gamma }}{R_{hkl}^{\gamma }}\right)}\times 100\%$$

Here, n and m refer to the number of *hkl* planes of γ and ferrite (α) or bainite or martensite, respectively. $I_{hkl}^{\gamma }$ and $I_{hkl}^{\alpha }$ are measured diffraction intensities whereas $R_{hkl}^{\gamma }$ and $R_{hkl}^{\alpha }$ are theoretical ones. It is recommended to use many *hkl* diffraction peaks. This correction method is however not satisfactory for engineering steels with rather strong texture. For such engineering steels, Gnäupel-Herold and Creuziger employed an angular dispersive neutron diffraction method using the 200, 220 and 311 peaks for γ and 200, 211 and 220 for α [26]. They have proposed that the measurements from more than 100 directions are necessary to avoid the texture influence. On the other hand, Xu et al. piled up thin square plates to make cubic specimens of TRIP steels with different *fγ* and studied this issue using a time-of-flight (TOF) neutron diffraction method [27]. The results obtained by such a measurement with the scattering vector parallel to the normal (ND), rolling (RD) or transverse (TD) direction of the steel plate were different from each other where the correction using Equation (1) was not sufficient. Hence, they measured TOF profiles from 525 directions in order to determine orientation distribution function (ODF) and *fγ* simultaneously employing the MAUD software [28,29]. Their conclusion has been reconfirmed in Ref [5] by comparing the results of neutron diffraction with those of transmission electron microscopy (TEM), electron backscatter diffraction (EBSD) and laboratory and synchrotron X-ray diffractions. This ex situ neutron diffraction measurement will be explained in Chapter 2.

The characterization of the retained γ during steel production is very important and hence many challenges have been made so far [22,23,25–27]. In most of such works, a specimen was interruptedly quenched during material processing in order to freeze its microstructure at a certain elevated temperature. It is, however, difficult to completely freeze high-temperature microstructure and thus not easy to track microstructure evolution continuously, or impossible to monitor the same observing position of a specimen. Hence, *in situ* observations and measurements of microstructural change during heat treatment have been expected to be realized by using various techniques. For example, *in situ* observations or measurements using confocal scanning laser microscopy [30–32], TEM [33,34], scanning electron microscopy (SEM)/EBSD [35], X-ray diffraction [36], etc. have been employed to track microstructure evolution during heat treatment. But it should be noted that the surface layer of a specimen is easily damaged at elevated temperatures even in vacuum or inert gas atmosphere: oxidation, decarburization, *etc*. hinder to obtain the information inside a sample. In some cases, *in situ* measurements using high energy synchrotron X-ray diffraction is a powerful tool for monitoring phase transformation [37–40]. In Chapter 3, I explain the results of dilatometry, *in situ* EBSD measurement, *in situ* X-ray diffraction and *in situ* neutron diffraction upon heating and subsequent cooling for a 1.5Mn-1.5Si-0.2C steel reported in Refs [6,7].

Phase transformation is affected by external and internal stresses that are yielded by misfit strains. In the case of ferritic transformation for a low C steel, transformation strains are expansive whereas C enrichment also occurs in the untransformed γ. The former brings tensile whereas the latter compressive elastic strains in the γ and therefore these two must compensate from each other resulting in decreasing of internal stress. Pearlitic transformation accompanies expansive transformation strains and misfit strains at semi-coherent α-cementite interface [41]. Internal stress distribution caused by these misfit strains is suspected to change the crystal orientation relationship between α and cementite resulting in a complex topology of pearlite structure [42]. In martensitic transformation, the misfit strains are composed of shear and dilatative strains with respect to the habit plane of a martensite lath, and the combination of multiple lath variants compensates internal stresses from each other, resulting in nearly hydrostatic tensile strains [43]. Nonetheless, the generation of compressive internal stresses in the retained γ was reported by X-ray diffraction experiments [44,45]. This is quite puzzling because martensite lath region expands resulting in hydrostatic compressive stresses whereas tensile in the matrix. Hence, to monitor the change in lattice spacings of the constituents directly during transformation is very important to understand the transformation mechanisms. Presently, applying the neutron diffraction technique to the investigation of martensitic transformation in a medium carbon steel, it would become possible to make clear real phase stresses paying attention that the lattice spacing is influenced not only by stress but also other influential factors. It is strongly expected directly to monitor microstructural evolution and internal stresses during steel production process, in particular, thermo-mechanically controlled processing (TMCP), which has been challenged using *in situ* neutron diffraction. This topic will be briefly reviewed in Chapter 4, the details of which will be reported in another paper [9].

## 2. Measurement of the volume fraction of the retained γ

**Figure 1.**
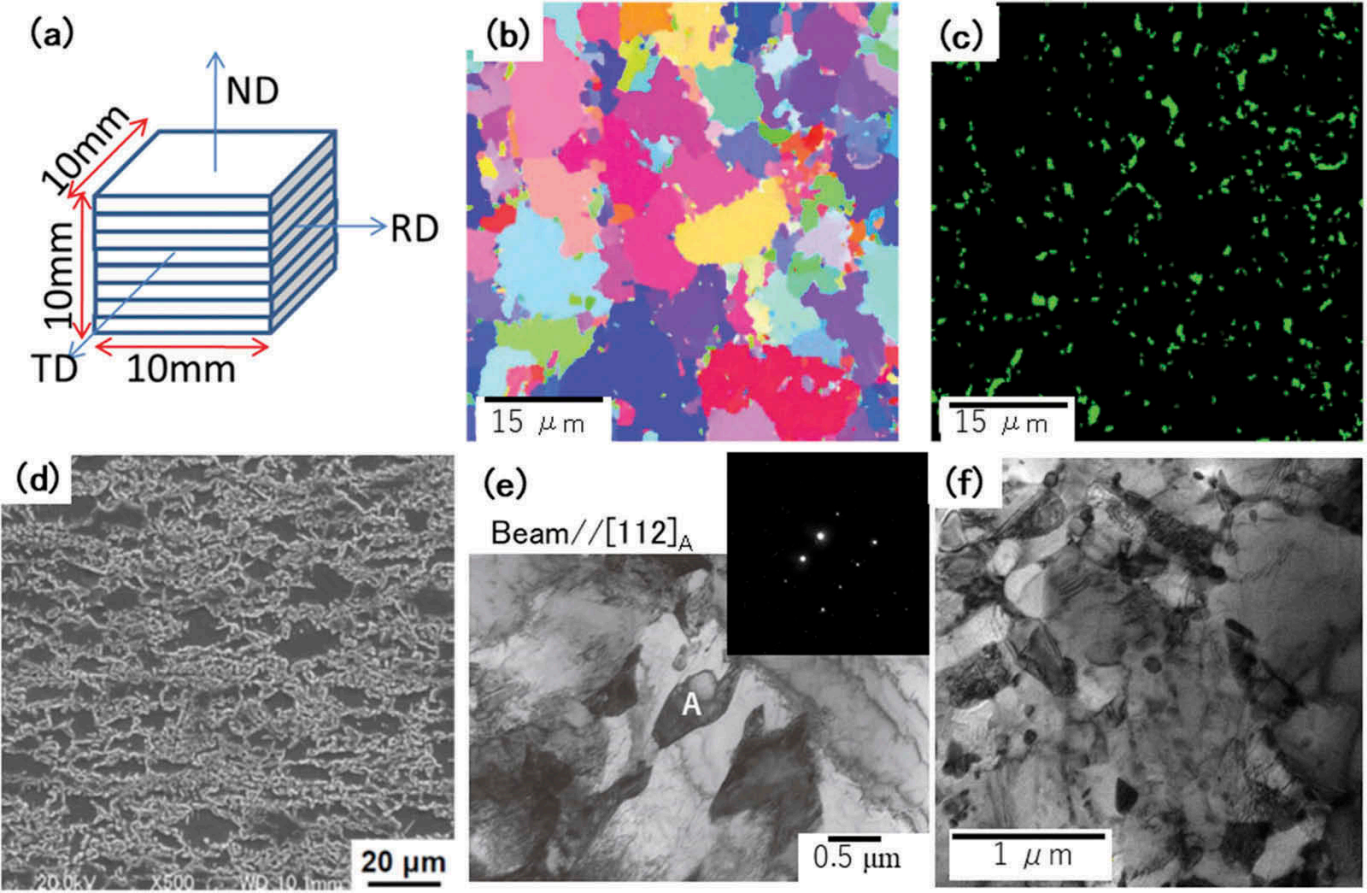
Schematic illustration explaining three directions of a studied steel sheet: ND, TD and RD (a) and microstructures: (b) inverse pole figure (IPF) map for TR2, (c) phase map for TR2 (green: γ), (d) SEM micrograph for TR2, (e) TEM microstructure for TR1 in which a γ grain is labeled by A and (f) TEM microstructure of TR3. Reproduced with permission from Tomota et al. [5–7]. Copyright 2017, 2018 Iron and Steel Institute of Japan.

A 1.5Mn-1.5Si-0.2C steel was melted referring to the work on TRIP steels made by Sugimoto et al. [46]. Hot-rolled plates were heat-treated with different conditions to prepare samples TR1, TR2 and TR3 as reported in Refs [5,6]. The final thickness of the steel sheet examined was 1.0 mm. These sheets were piled up to be a cuboidal specimen of 10 × 10 × 10 mm^3^ for neutron diffraction as shown in Figure 1(a), in which the specimen directions were defined as normal (ND), transverse (TD) and rolling (RD) directions. Similar samples to measure along these three directions were prepared for laboratory and synchrotron X-ray diffraction measurements (RINT-2500H/PC; Rigaku Co., Japan and SPring8 BL15XU, Japan, respectively). The inverse pole figure (IP) map, phase map and SEM microstructure (JSM-7000F; JEOL Co., Japan) of TR2 are presented in Figure 1(b–d), respectively. The SEM microstructures of samples TR1, TR2 and TR3 observed with a low magnification are very similar. A typical example is presented in Figure 1(d) which consists of mainly α phase and bainite. The α grain size was approximately 10 μm. As observed in Figure 1(e,f), the retained γ grains were found by TEM in TR1 and TR2 but not in TR3 in which cementite particles were detected instead of γ. The statistically reliable measurement of *fγ* by TEM observation (JEM-200FX and 2010F; JEOL Co., Japan) is extremely difficult. In some α grains, dislocations were observed (the density of 10^14^ m^−2^ order). The *fγ* determined by EBSD (TSL SC-200; TSL solutions K.K., Japan) is affected by the measuring area size, magnification and image cleaning technique. The measurements with the magnification of 500 times, 100 × 100 μm^2^ scanning area and 0.1 μm scanning pitch showed 4.1% (TR1), 4.7% (TR2) and less than 0.5% (TR3). Smaller values of *fγ* for TR1 and TR2 compared with the results by other methods are suspected to stem from the stability of γ. That is, the result is very sensitive to specimen preparation procedure.

**Figure 2.**
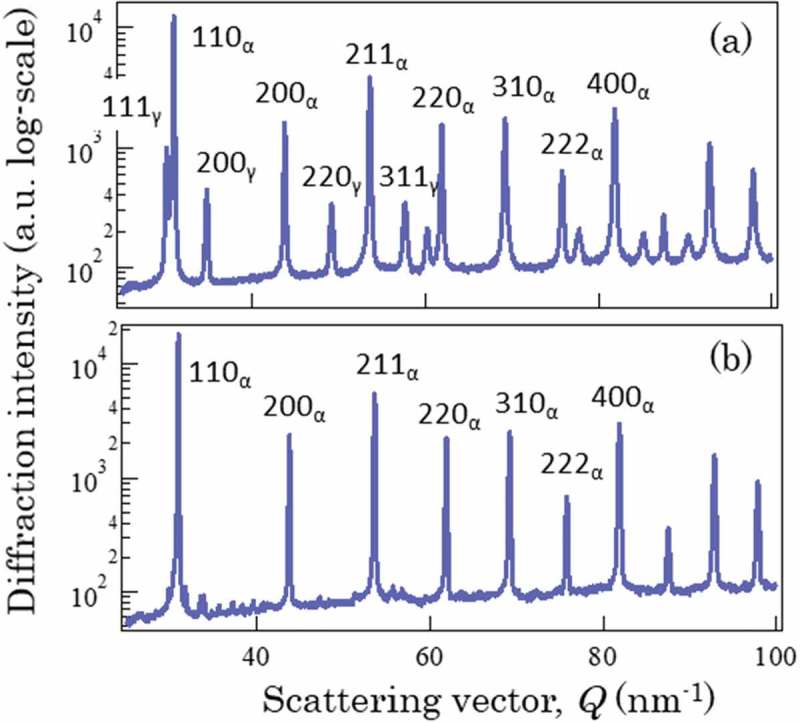
Neutron diffraction profiles obtained for TR1 and TR3 (TD), in which the intensity was plotted in a log-scale. Reproduced with permission from Tomota et al. [5]. Copyright 2017 Iron and Steel Institute of Japan.

Neutron diffraction was performed at BL19 of MLF J-PARC [47,48]. Figure 2 depicts the neutron diffraction profiles for TR1 (a) and TR3 (b) measured from TD. To highlight low-intensity peaks clearly, the log-scale vertical axis was employed. On the other hand, the magnitude of scattering vector,Q defined by $\frac{4\pi sin\theta }{\lambda }\left(=\frac{2\pi }{d}\right)$was adopted for the horizontal axis where θ, λ, and d refer to a half of diffraction angle, wavelength and lattice plane spacing, respectively. As seen, γ diffraction peaks are apparently detected in (a) but not in (b) where the cementite diffraction peaks can be found. Similar results were obtained by laboratory and synchrotron X-ray diffractions (see Figures 5 and 7 in ref [5]). Synchrotron X-ray diffraction was carried out at BL15XU of SPring-8 [49].

The X-ray diffraction intensities are estimated by the following equations [50].

(2) $$I_{hkl}=\left\{I_{0}e^{4}\lambda^{3}A_{s}/\left(64\pi rm^{2}c^{4}\mu \right)\right\}R_{hkl}$$

(3) $$R_{hkl}={\left|F_{hkl}\right|}^{2}j_{hkl}\nu_{p}^{-2}L_{hkl}\cdot exp\left(-2M_{hkl}\right)$$

where *I*_hkl_: diffraction intensity, *I*_0_: incident X-ray beam intensity, *e*: charge of electron, *m*: mass of electron, *λ*: wavelength, *c*: light speed, *A*_s_: specimen area, μ: mean absorption coefficient, *r*: optical radius of system, *F*_hkl_: structure factor, *j*_hkl_: multiplication, *v*_p_: volume of unit cell, *L*_hkl_: Lorenz polarization factor, exp(−2*M*_hkl_): thermal factor. Inputting the result of Equation (2) and measured intensity into Equation (1) assuming *R*_α_/*R*_γ_ = 1.37, *fγ* was computed and the results by laboratory X-ray diffraction are tabulated in Table 1. As is found, the calculated results depend on the measuring direction both in TR1 and TR2, indicating the correction by Equation (1) is not satisfactory. The results by synchrotron X-ray diffraction and neutron diffraction are also listed in Table 1. In the case of neutron diffraction, the hkl diffraction intensity of a texture-free steel was calculated by the Z-Rietveld software. To be noted here is the *fγ* determined from the ND direction is lower than those from the TD and RD, commonly in laboratory X-ray, synchrotron X-ray and neutron diffractions (the ND results were highlighted by blue characters). Synchrotron X-ray diffraction showed the highest resolution, but its beam size was too small to obtain bulk-averaged value. In contrast, the gauge volume of neutron diffraction is 5 × 5 × 5 mm^3^, so that reflection beams from several million grains merge together, which is preferable to obtain the global averaged information. In neutron diffraction, a specimen rotation using a Gandolfi camera was employed, but it was found insufficient to remove the texture influence.

**Table 1.** Austenite volume fraction (vol. %) determined by different methods, in which the data for ND are highlighted by blue characters.

	TR1	TR2
Neutron		
Texture^a^	14.8	13.9
Average^b^	14.6	13.8
Gandolfi^c^	16.3	15.3
ND	11.3	13.8
RD	13.0	14.2
TD	15.0	14.1
Synchrotron		
ND	10.9	9.0
RD	13.6	12.6
TD	15.2	15.9
Labo. X ray		
ND	12.2	12.7
RD	13.7	13.4
TD	14.3	15.4
EBSD	6.4	5.5

^a^Simultaneous measurement with texture (ODF).

^b^Average of the results from 525 directions.

^c^Measurement using a Gandolfi camera.

**Figure 3.**
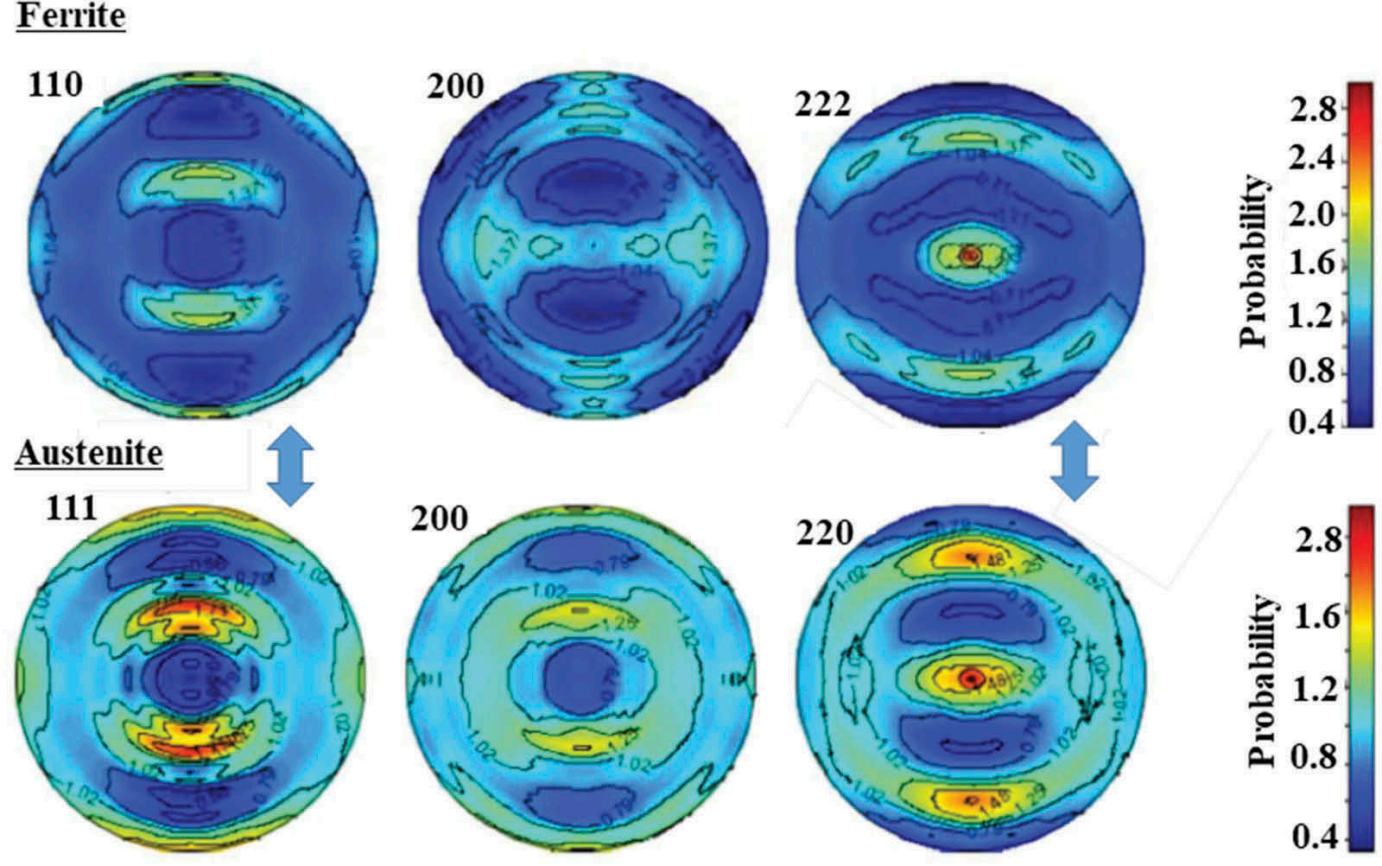
Main *hkl* pole figures for α and γ phases obtained by neutron diffraction, where the arrows indicate the K-S relationship between body-centered cubic (bcc) and face-centered cubic (fcc) phases. Reproduced with permission from Tomota et al. [5]. Copyright 2017 Iron and Steel Institute of Japan.

**Figure 4.**
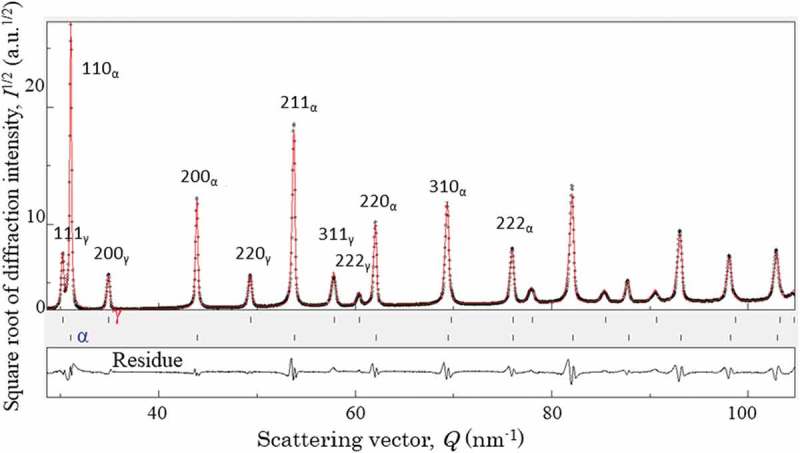
Results of MAUD analysis for TR1, where the intensity in square root was plotted to emphasize the fitting residue clearly. Reproduced with permission from Tomota et al. [5]. Copyright 2014 Iron and Steel Institute of Japan.

Partial pole figures were measured with the X-ray Schultz method whereas whole pole figures with neutron diffraction. The results obtained by EBSD, X-ray and neutron for TR1 and TR2 were similar (see Figures 6 and 11 in ref [5]). Figure 3 shows the results for TR1 obtained by neutron diffraction suggesting that the K-S relationship between α and γ [51] holds (see arrows). Consequently, such a texture hinders to get the true *fγ* and therefore simultaneous determination of *fγ* and ODF has been proposed [5,27]. Then, Euler cradle was employed for the cuboidal specimen shown in Figure 1(a) for neutron diffraction measurement. TOF diffraction profiles were gathered from 525 directions and the obtained data were input into an ODF calculation software, MAUD [28,29]. The pole figures obtained by this method are presented in Figure 3. The fitting result for TR1 to determine *fγ* is presented in Figure 4 as an example and finally obtained values of *fγ* were 14.8% for TR1 and 13.9% for TR2. When the diffraction profiles obtained from 525 directions were simply summed as Gnäupel-Herold and Creuziger proposed [26] and analyzed using Z-Rietveld software [52,53], the values of 14.6% were obtained for TR1 and 13.8% for TR2 showing good agreements with the MAUD results. If the measurement has to be limited only from one direction, the TD direction would be recommended as was discussed by Järvineng [54].

With respect to the obtained neutron diffraction profiles, the convolutional multiple whole profile (CMWP) fitting method [55,56] was applied resulting in the dislocation densities of 0.982 × 10^14^ m^−2^ for TR1 and 1.13 × 10^14^ m^−2^ for TR2, roughly supporting the TEM observations described above.

**Figure 5.**
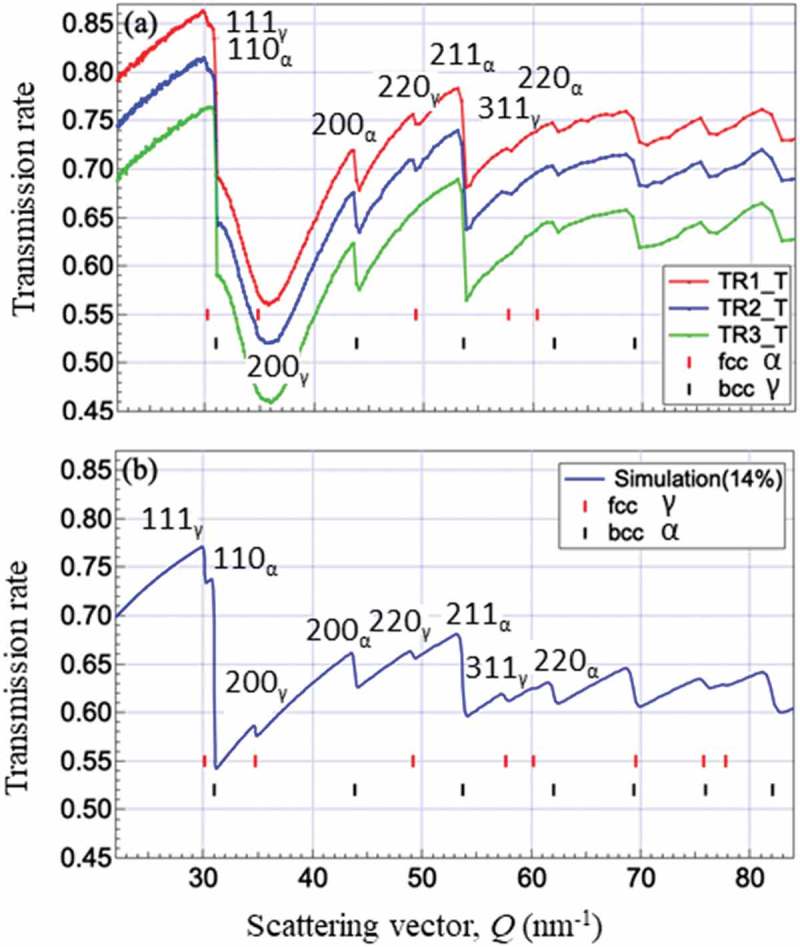
Transmission neutron Bragg-edge spectra for TR1, TR2 and TR3 (a) and simulation result for texture-free specimen with $f_{\gamma }$= 0.14. Reproduced with permission from Tomota et al. [5]. The wavelength was used for horizontal axis in the original paper. Copyright 2017 Iron and Steel Institute of Japan.

The retained γ was also characterized by small-angle neutron scattering [57] and transmission Bragg-edge measurement. Although the space-resolution of the latter technique is sub-millimeter order at the present, it is expected to inspect the integrity of mechanical components with a compact neutron source. Woracek et al. [58] showed a three-dimensional distribution of deformation-induced martensite in a specimen subjected to tension or torsion. Hereby to examine such an application, transmission Bragg-edge spectra were obtained at BL22 MLF J-PARC [59] and the obtained results are presented in Figure 5. In the transmission Bragg-edge measurement, transmission ratio is usually plotted as a function of neutron wavelength, but here the scattering vector, $Q\left(=\frac{4\pi }{\lambda }\right)$, was employed ($\sin\left(90^{\circ }\right)=1.0$) in order to compare it with the above diffraction results. As seen, though γ Bragg edges for 111, 220 and 311 were found in TR1 and TR2, that for 200 was hardly recognized because of strong texture. An ideal Bragg-edge spectrum for a texture-free steel was calculated by a Rietveld like fitting software, RITS developed by Sato [60] assuming *fγ* = 14％ and the result is presented in Figure 5(b). It would be understood that more improvement is required for the determination of *fγ* by this unique technique.

In conclusion, the determined amount of *fγ* depends on measuring method generally showing the trend to become larger, EBSD, X-ray and neutron in order. The simultaneous determination of *fγ* and ODF using neutron diffraction is recommended.

## 3. Decomposition of the retained γ and reverse γ transformation upon heating

**Figure 6.**
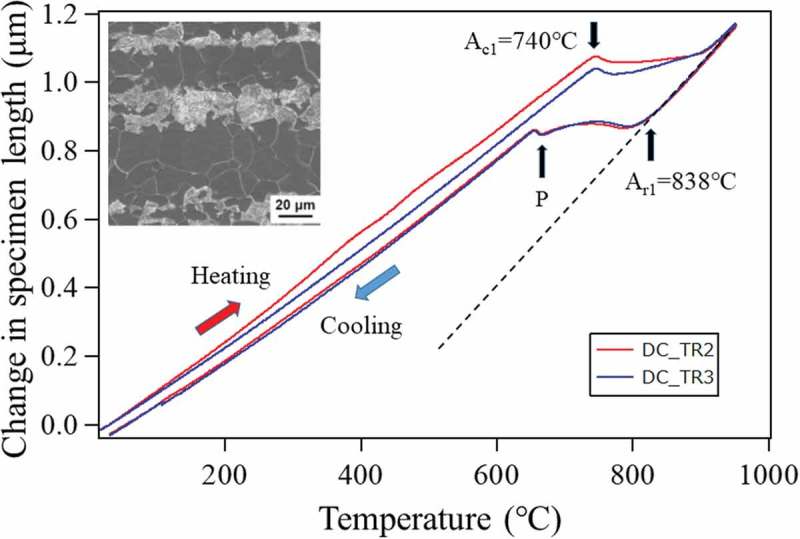
Dilatometry curves for TR2 and TR3 in which SEM microstructure after cooling was inserted. Reproduced with permission from Tomota et al. [6,7]. Copyright 2017, 2018 Iron and Steel Institute of Japan.

The dilatometry curves on heating and cooling with a constant speed of 5°C/s for TR2 and TR3 are presented in Figure 6. The two curves are similar except at the temperature region around 300– 400°C upon heating, where the decomposition of the retained γ to α and cementite phases accompanying expansion is observed in TR2 but not in TR3. These two specimens show almost identical reverse transformation temperature, i.e. A_c1_ (= 740°C), indicating that the microstructures just before the onset of reverse transformation are similarly composed of α and cementite phases. Inserted in Figure 6 is the microstructure of TR2 at room temperature (RT) after the test which consists of α and pearlite suggesting the Mn bands formed during cooling. The volume fraction of pearlite determined by SEM was approximately 26%.

**Figure 7.**
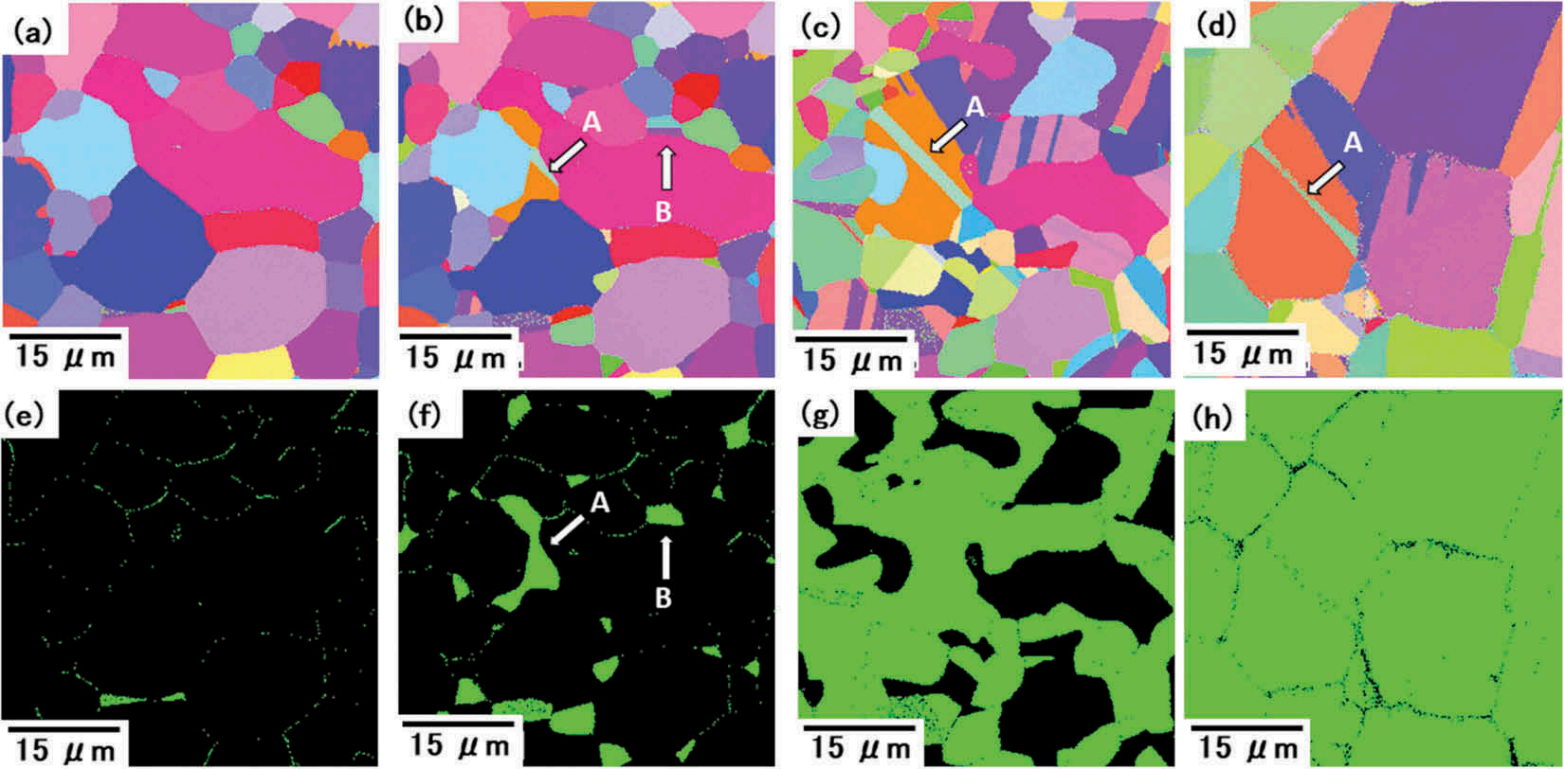
Reverse γ transformation behavior observed with *in situ* EBSD for TR2: (a)–(d) IPF maps, (e)–(h) phase maps, (a), (e) 924°C (γ volume fraction: 0.009), (b), (f) 940°C (0.083), (c), (g) 964°C (0.640), and (d), (h) 997°C (0.982). Reprinted with permission from Tomota et al. [6]. Copyright 2017 Iron and Steel Institute of Japan.

*In situ* EBSD observations (HSEA-1000; TSL solutions K.K., Japan) on the surface during heating were carried out for TR2. The specimen was heated quickly up to A_c1_ temperature and then slowly in order to observe the reverse transformation behavior. Some γ grains appeared at 924°C (see Figure 7(a,e)), which is much higher than A_c1_ determined by dilatometry in Figure 6. The growth of some γ grains containing annealing twin labelled A is identified in Figure 7(b,f). On the other hand, another grain labelled B in (b) and (f) shrinks with further annealing. At 997°C in (d) and (h), the surface of the specimen was covered almost completely by γ grains. Since the γ grain nucleation is considered to take place not only at the surface but also interior of the specimen, it is not suitable to monitor the microstructural change on the surface to elucidate the nucleation and growth behavior of the γ reverse transformation. Very serious problems of this *in situ* EBSD observation include that transformation temperatures were about 200°C higher than those determined by the conventional dilatometry test.

**Figure 8.**
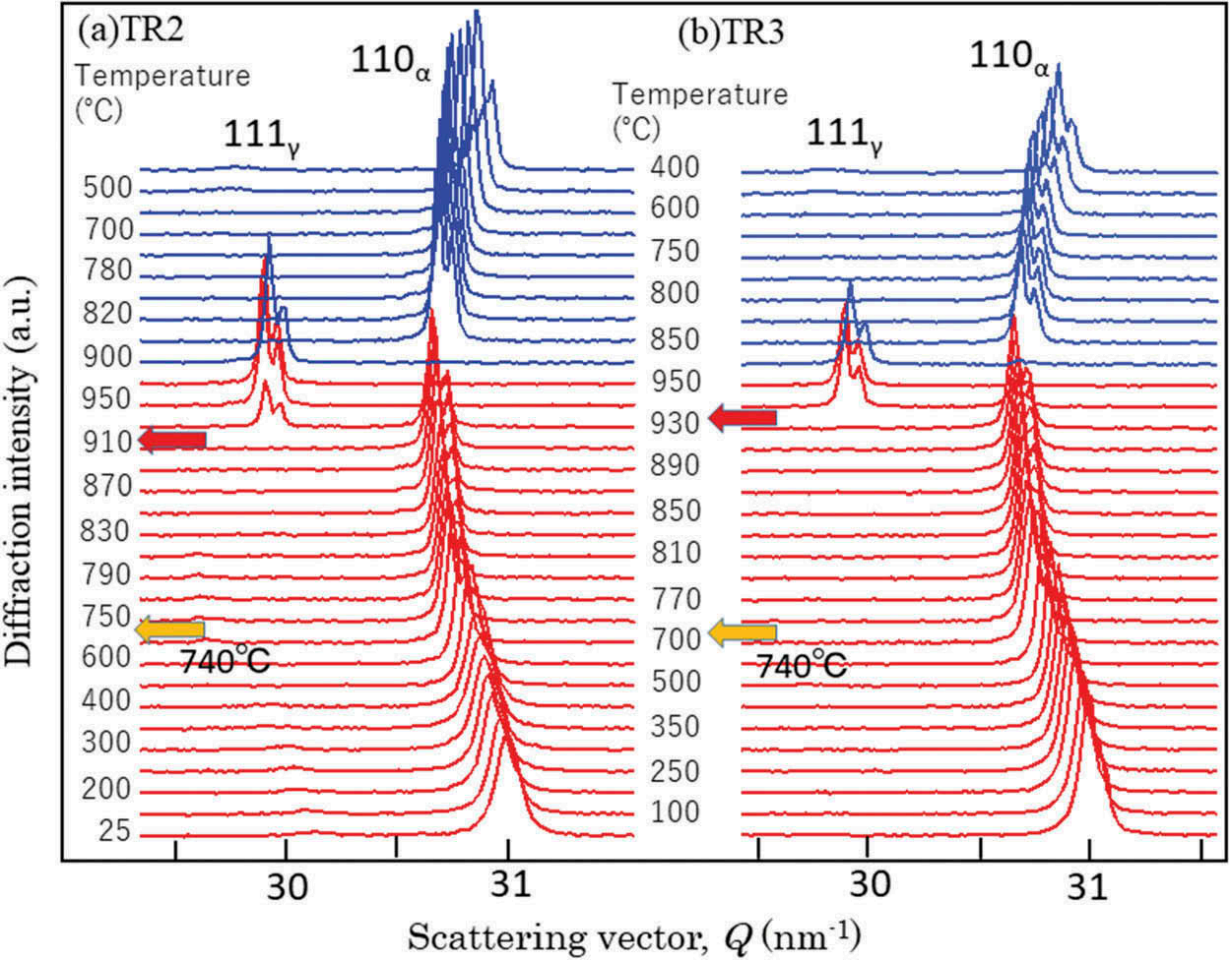
Changes in γ 111 and α 110 X-ray diffraction peaks during heating and cooling: (a) TR2 and (b) TR3. Reproduced with permission from Tomota et al. [6]. Copyright 2017 Iron and Steel Institute of Japan.

Figure 8 depicts the results of *in situ* X-ray diffraction (Multi-purpose X-ray diffractometer, Spectris Co. Ltd., UK) with heating and cooling where the changes in 110 α and 111 γ peaks are drawn. The 111γ diffraction peak was observed at 25°C in (a) for TR2 but not in (b) for TR3. Concerning the γ reverse transformation, the γ peaks did not appear until temperature increased up to 930°C, much higher than A_c1_ (740°C) determined by the dilatometry test, similarly to the case of *in situ* EBSD measurement. It is suspected the specimen surface would be modified during these measurements.

**Figure 9.**
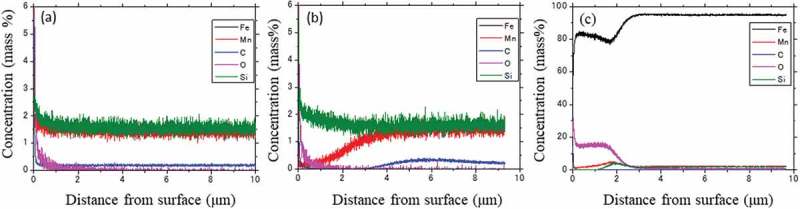
Chemical compositions as a function of the depth from surface obtained by RF glow discharge optical emission spectroscopy for the EBSD specimens before heating (a) and after elevated temperature measurements of EBSD (b) and X-ray diffraction (c). Reproduced with permission from Tomota et al. [6,7]. Copyright 2017, 2018 Iron and Steel Institute of Japan.

To elucidate the reasons why transformation temperatures determined by EBSD and X-ray diffraction were higher than those by dilatometry, the chemical compositions were inspected using radio-frequency (RF) glow discharge optical emission spectroscopy (RF GDOES: HORIBA GD-Profiler 2) with serial removal of the surface layer. The obtained results are depicted in Figure 9, in which panels (a), (b) and (c) were obtained from a non-heated specimen, after *in situ* EBSD measurement and after in situ X-ray diffraction, respectively. As seen in panels (b) and (c), Mn and C concentrations are lowered in the region from the surface to approximately 3 μm in depth. The chemical compositions examined at the surface by energy-dispersive X-ray spectroscopy (EDS) analysis have also revealed that C and Mn contents were lowered after heating for *in situ* EBSD measurement. Therefore, in addition to decarburizing, Mn atoms must easily be desorbed from the surface in a high vacuum due to its higher equilibrium vapor pressure. If Mn and C concentrations decreased, i.e. Fe-Mn-Si-C alloy changed to Fe-Si alloy, α phase would become more stable, resulting in higher transformation temperatures. The transformation temperatures for these chemical compositions computed using Thermo-Calc TCFE6 supported well this conclusion [6]. Interestingly, the C concentration profiles in Figure 9(b,c) exhibit a slight increase in the interior. The C diffusion must take place towards the specimen inside during α transformation started from the surface on cooling, as was already reported [61,62].

In general, quantum beam intensity $I\left(x\right)$ decreases with penetrating path length following Equation (4).

(4) $$I\left(x\right)=I_{0}exp\left(-\mu x\right)$$

Here, $I_{0}$, μ and *x* refer to the incident beam intensity, linear absorption coefficient and the length of flight path, respectively. According to Wisniewski and Russel [63], the depth of the effective information volume of EBSD performed with 20 kV is a few hundred nanometers for steel. If we employ the μ-values of 257 mm^−1^ for the X-ray beam and 0.012 mm^−1^ for neutron [64], the values of *x* where the intensity ratio of diffraction beam to the incident one becomes 1/*e* (= 1/2.7182), would be 0.00389 mm for X-ray and 83 mm for neutron. Therefore, neutron is superior to the other beams for this kind of purpose.

**Figure 10.**
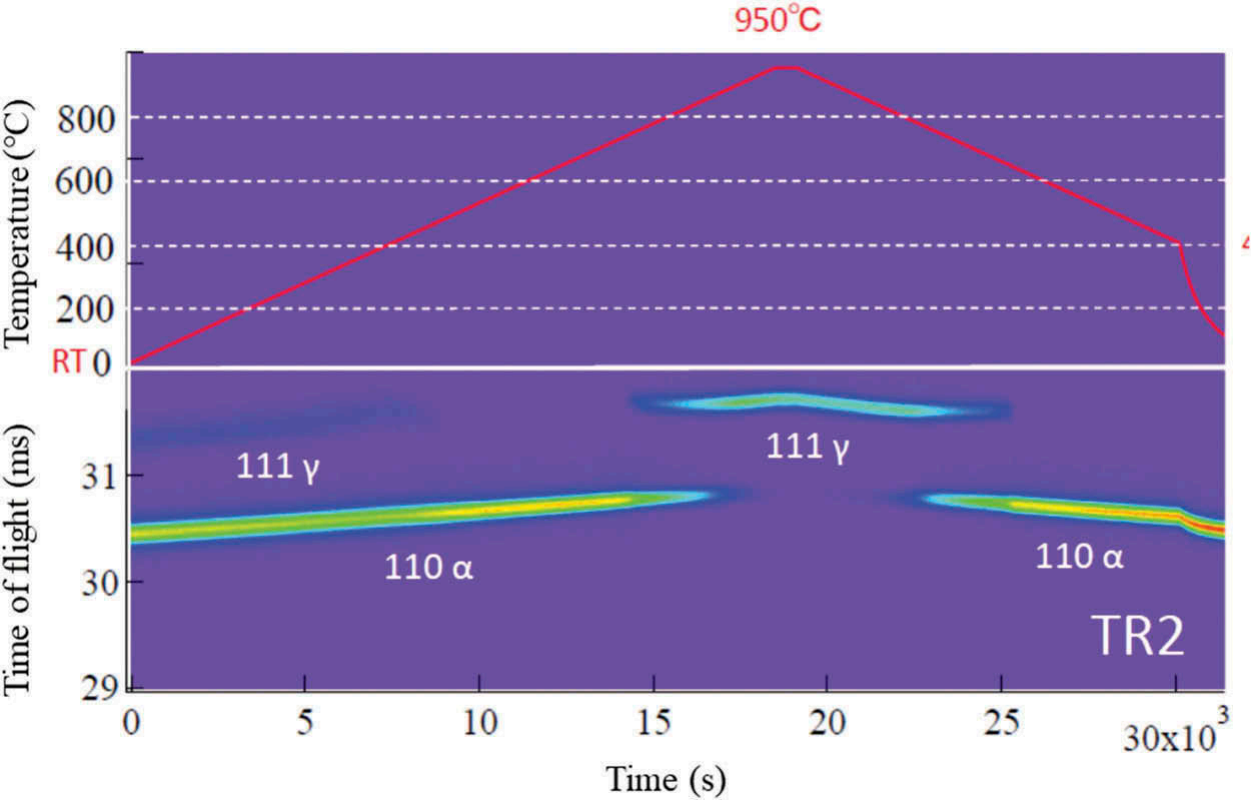
Changes in neutron diffraction intensities for 111 γ (γ) and 110 α (α) upon heating for TR2. Reprinted with permission from Tomota et al. [6]. Copyright 2017 Iron and Steel Institute of Japan.

**Figure 11.**
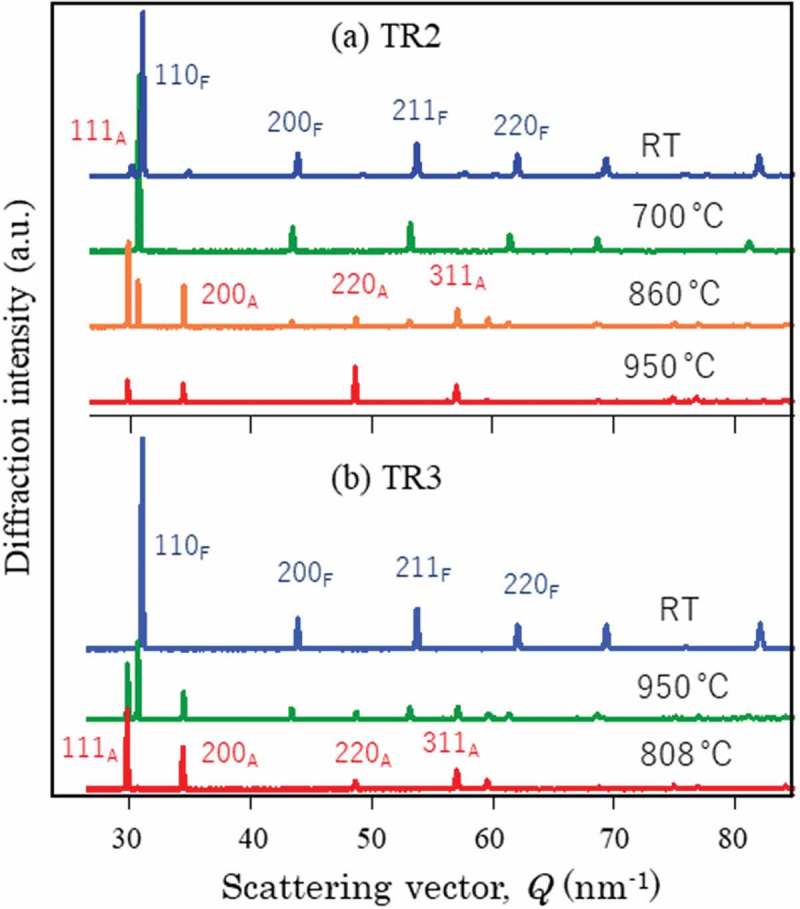
Neutron diffraction profiles obtained at various temperatures: (a) TR2 and (b) TR3. Reproduced with permission from Tomota et al. [6]. Copyright 2017 Iron and Steel Institute of Japan.

**Figure 12.**
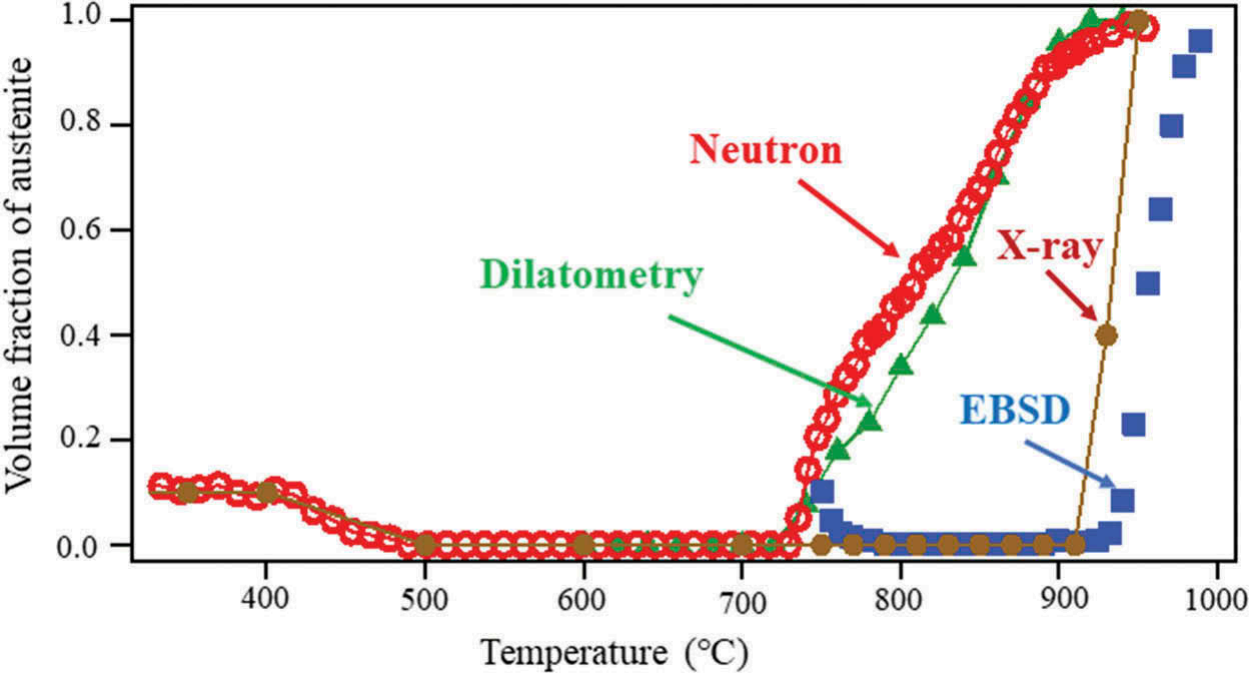
Changes in γ volume fraction as a function of temperature determined by the four different methods for TR2. Reproduced with permission from Tomota et al. [6]. Copyright 2017 Iron and Steel institute of Japan.

**Figure 13.**
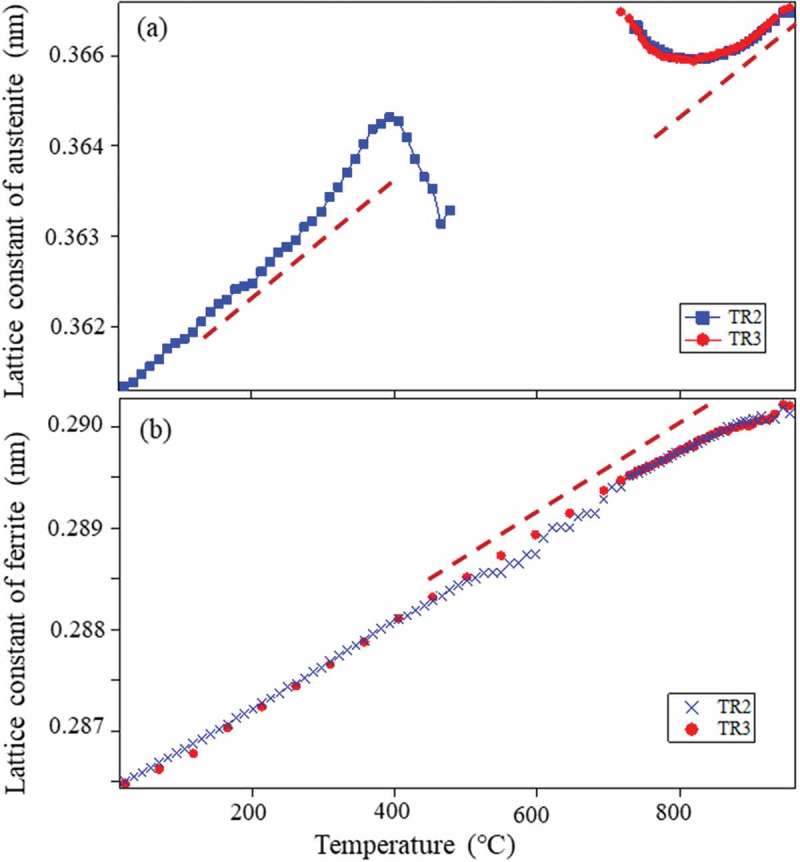
Changes in the γ and α lattice constants determined by neutron diffraction with heating for TR2 and TR3. The dashed lines refer to the thermal expansion of γ or α estimated from the temperature regions for their constant chemical compositions. Reprinted with permission from Tomota et al. [6]. Copyright 2017 Iron and Steel Institute of Japan.

Changes in 111 γ and 110 α diffraction peaks observed by *in situ* neutron diffraction during heating and cooling are presented in Figure 10. The whole diffraction profiles at typical temperatures are depicted in Figure 11. As seen, γ exists in TR2 but not in TR3 at RT. The decomposition behavior of the retained γ is clearly confirmed to occur from 400°C to 500°C for TR2. In Figure 12, the *fγ* calculated by the Z-Rietveld software was plotted as a function of temperature. As was discussed above, the result obtained in the RD direction was higher than that in the ND direction, but only the results in the RD direction were plotted in Figure 12 for simplicity (detailed discussion was given in Ref [6]). Important is that the onset of γ reverse transformation is found near 740°C, showing an excellent agreement with the result of dilatometry. This is because neutron diffraction enables us to monitor the bulky averaged information hardly influenced by surface damage. However, being different from dilatometry measurement, *in situ* neutron diffraction measurement provides us more fruitful insights on microstructure change. As examples, lattice parameters of α and γ were determined using the Z-Rietveld software and the obtained results are presented in Figure 13. During heating, the decomposition of retained γ and reverse γ transformation occurred. It is found in (a) that the γ lattice parameter increases with increasing of temperature due to thermal expansion. Around 400°C, the deviation from the thermal expansion line is observed. Most likely mechanism of decomposition of the retained γ predicted from the present results would be the increase of C concentration of γ with α formation, followed by the precipitation of cementite accompanying the decrease in *fγ* as well as the decrease in C concentration in γ leading to ferritic transformation.

At the reverse γ transformation, the nucleation site of γ grain is postulated to be α/cementite interface and therefore the C concentration of γ must be higher caused by dissolution of cementite particles. The high C bearing γ is also predicted from the tie line of phase diagram. Hence, the γ lattice parameter deviates from the thermal expansion line as is observed in Figure 13(a). With increase in *fγ*, the γ lattice parameter approaches to its thermal expansion line. Here, the α lattice parameter also deviates towards lower values from its thermal expansion line, indicating the generation of hydrostatic compressive internal stresses caused by the expansive transformation strains. The deviation of γ lattice parameter must include the influence of hydrostatic tensile stresses which are balanced with those in the α phase. In conclusion, neutron diffraction is superior to study microstructure evolution at high temperature compared with dilatometry, laser microscopy, electron microscopy and X-ray diffraction but only the global information can be obtained. Therefore, neutron diffraction should be complementarily used with other techniques like EBSD to connect macroscopic and microscopic insights to deepen the understanding. If the influence of surface damage can be neglected, it would become a very powerful method [65].

## 4. Ferritic and pearlitic transformations upon cooling

Phase transformation behavior from γ during cooling for the above 1.5Mn-1.5Si-0.2C steel was *in situ* monitored using dilatometry, X-ray diffraction and neutron diffraction. As was presented in Figure 6, the change in specimen length on cooling is found to deviate from thermal contraction curve, indicating the occurrence of phase transformations. Here, thermal expansion coefficients of α ($K^{\alpha }$) and γ ($K^{\gamma }$) were determined to be $13.5\times 10^{-6}{/}^{{}^{\circ}}\text{C}$ and $21.0\times 10^{-6}{/}^{{}^{\circ}}\text{C}$, respectively. Upon cooling from 950°C, the deviation from the thermal contraction line of γ was found nearly at 838°C indicating the start of ferritic transformation, i.e. Ar_3_. This deviation is attributed to the expansive strains of γ to α transformation. Under the constant cooling rate of 0.05°C/s, transformation speed was higher in the beginning, then became lower and again higher after the point P. This retardation of the transformation is believed to stem from C and Mn diffusions in the untransformed γ and the point P is likely to indicate the onset of pearlitic transformation.

**Figure 14.**
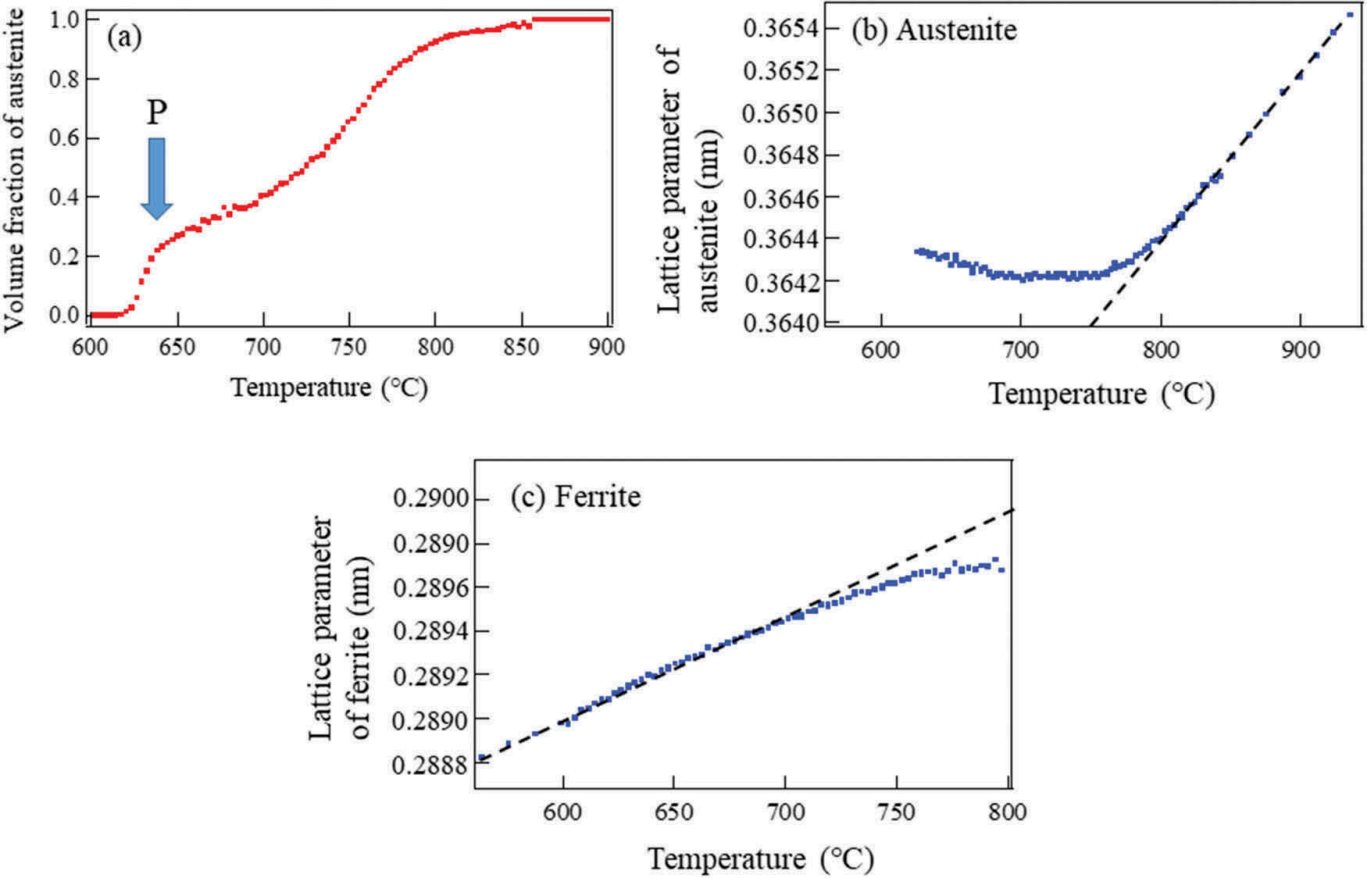
Change in γ volume fraction (a), γ lattice parameter (b) and α lattice parameter (c) during cooling determined by in situ neutron diffraction measurement. Reproduced with permission from Tomota et al. [6]. Copyright 2017 Iron and Steel Institute of Japan.

Changes in 111γ and 200γ and 110α peaks obtained by *in situ* neutron diffraction during cooling are presented in Figure 10 together with sample temperature as a function of elapsed time. As is observed in Figure 14(a), the $f_{\gamma }$ decreases rapidly in the beginning, then slowly and again rapidly after the point P with decreasing of temperature. Here, the start temperature of ferritic transformation shows a good agreement with the result of dilatometry, similarly to the above heating results. Again, being different from dilatometry, *in situ* neutron diffraction provides more fruitful insights on microstructural change. The lattice parameters of α and γ were determined using the Z-Rietveld software and the obtained results were presented in Figure 14(b,c), respectively. The γ lattice parameter depends on temperature$\;\left(T\right)$, C concentration$\;(C\left(mass\%\right)$) and elastic internal strain$\;\left(\varepsilon \right)$, i.e. $\;a^{\gamma }\left(T,C,\varepsilon \right)$. Similarly, the α lattice parameter is described as $a^{\alpha }\left(T,C,\varepsilon \right)$ although the C content is very limited. Thermal contraction could be expressed by a linear function. In the case of the γ region from 840°C to 940°C,

(5) $$a^{\gamma }\left(T,0.20,0\right)=\left(0.3580\pm 0.0005\right)+\left(7.991\pm 0.006\right)\times 10^{-6}T\;\left(nm\right)$$

and in the case of the α region from 550°C to 610°C,

(6) $$a^{\alpha }\left(T,0,0\right)=\left(0.2863\pm 0.0009\right)+\left(4.479\pm 0.008\right)\times 10^{-6}T\;\left(nm\right)$$

Thermal expansion coefficient was calculated using the lattice parameter of 0.3646 nm at 820°C for γ$\;\left(K^{\gamma }\right)$ and 0.2889 nm at 575°C for α$\;\left(K^{\alpha }\right)$ and the obtained results were $21.9\times 10^{-6}{/}^{{}^{\circ}}C$ and $15.5\times 10^{-6}{/}^{{}^{\circ}}C$, respectively. These are in good agreement with those determined by dilatometry. The obtained $K^{\gamma }$ is also close to the values reported by Onink et al. [66] and Bhadeshia et al. [67]. According to Onink et al.’s results by neutron diffraction experiments for Fe-C binary alloys, the value of $K^{\gamma }$ decreases slightly from $24.7\times 10^{-6}$to $22.9\times 10^{-6}{/}^{{}^{\circ}}\text{C}$ with increasing of C content from 0.01 to 0.8 mass%.

The α lattice parameter deviates from its thermal contraction line in Figure 14(c), suggesting that compressive internal stress generated due to the expansive transformation strains. In contrast, the γ lattice parameter deviates towards upper side in Figure 14(b). It must be due to the internal stresses balanced with those in α and another important origin for this deviation would be C enrichment [68,69]. The deviation from the thermal contraction line is, therefore, attributed to ① transformation strains, ② thermal misfit strains and ③ C enrichment in γ.

The γ→α transformation strains depend on temperature. At 740°C where approximately 50% transformation had been progressed, the volume per Fe atom calculated by $V^{\gamma }=\frac{0.36370^{3}}{4}=0.012027\left(nm^{3}\right)$ for γ and $v_{a}=\frac{0.28961^{3}}{4}=0.012145\left(nm^{3}\right)$ for α would result in the volume strain of γ to α transformation of 0.981%. Assuming isotropic expansion, transformation strains ($\varepsilon_{ij}^{trans}$) are described as $\varepsilon_{11}^{trans}=\varepsilon_{22}^{trans}=\varepsilon_{33}^{trans}=0.327\%$. If one computed transformation strains using extrapolated parameters at 0°C, $\varepsilon_{v}$ would become 2.3% and then $\varepsilon_{11}^{trans}=0$.767%. As will be discussed later, $\varepsilon_{ij}^{trans}$ is also affected by C concentration of γ which increases with γ→α transformation leading to lower the value of $\varepsilon_{ij}^{trans}$.

In a γ-α two-phase temperature region, thermal misfit strain ($\varepsilon_{ij}^{ther}\left(T\right)$) generates due to the difference in thermal expansion coefficients between γ and α, which is −6.4 × 10^−6^ (/°C). The thermal history is dependent on elapsing time after α forms and it could be obtained by

(7) $$\begin{array}{rl} & \varepsilon_{ij}^{ther}\left(T\right)=\int_{T}^{838}f_{a}\left(T\right)\times \left(-6.4\times 10^{-6}\right)\times \left(838-T\right)dT/\\ & \;\;\;\;\;\;\;\;\;\;\;\;\;\;\;\;\;\;\;\;\;\;\;\;\;\int_{T}^{838}fa\left(T\right)dT \end{array}$$

where $f_{\alpha }\left(T\right)$ refers to α volume fraction. As described above, the start temperature of α transformation (Ar_3_) is 838°C. Then, assuming $f_{\alpha }\left(T\right)=constant\times \left(838-T\right)$, $\varepsilon_{ij}^{ther}\left(T\right)$ would roughly be estimated as $-6.4\;\times \;10^{-6}\times \frac{2\times \left(838-T\right)}{3}$ from Equation (6). In case of 740°C, $\varepsilon_{ij}^{ther}\left(740^{{}^{\circ}}\;C\right)=-6.4\times 10^{-6}\times \frac{2\times 98}{3}=-4.18\times 10^{-4}$if i = j (zero in other cases). The estimated $\varepsilon_{11}^{ther}\left(740^{{}^{\circ}}\;C\right)$ is about 0.04% which is one order smaller than $\varepsilon_{11}^{trans}$ value of 0.327%. Therefore, the below discussion must be acceptable even if the detailed calculation using the real $f_{\alpha }\left(T\right)$-T relation was skipped.

Several empirical equations related to the effect of C concentration on γ lattice parameter at RT have been proposed [70–73]. Here, the next relationship [73] is adopted.

(8) $$a^{\gamma }\left(RT,C,0\right)=a^{\gamma }\left(RT,0,0\right)+0.0033C\left(mass\%\right)=0.3573+0.0033C\left(mass\%\right)$$

Inputting RT = 20°C into Equation (5), $a^{\gamma }\left(RT,0.2,0\right)$ would be 0.35815 nm and then $C\left(mass\%\right)=$0.258 mass% by Equation (8). It must be acceptable taking into consideration the use of the empirical equations and ambiguous estimating procedure. Next, adopting the same thermal expansion coefficient for γ with different C content and the lattice parameter at 620°C, $a^{\gamma }\left(620,C,0\right)$ = 0.36430 nm, the lattice parameter at RT, $a^{\gamma }\left(RT,C,0\right)$, could be estimated to be 0.35951 nm and thus the C concentration of 0.667 mass%. On the other hand, the pearlite volume fraction obtained by SEM microscopy was 26%. If the C concentration in α and grain boundary segregation were neglected, the C concentration in γ could be calculated as 0.20/0.26 = 0.763 mass% where 0.20 mass% is the bulk average. Therefore, pearlitic transformation would start when C concentration of γ is enriched to approximately 0.76 mass%. This seems to be reasonable in comparison with the point P in Figure 14(a).

The lattice expansion strain due to carbon enrichment $\varepsilon_{ij}^{C}$ was evaluated as $\varepsilon_{ij}^{C}=\frac{0.0033\times C\left(mass\%\right)}{a^{\gamma }\left(RT,0,0\right)}$. In the case of 0.7 mass% C, $\varepsilon_{ij}^{C}$ becomes 0.647%, which is also a kind of eigen strain to induce internal stresses.

If one estimated the γ fraction at P in Figure 6 employing the so-called lever rule for the drawn thermal contraction lines of α and γ, it would be about 10% which is much smaller than 26% determined from microstructure. This is because the adopted thermal contraction line is for γ with 0.2 mass% C not 0.76 mass% C. With increasing of C concentration in γ, its thermal contraction line shifts towards the upper side [66], leading to the estimation of higher *fγ*. This means the exact phase fraction cannot be determined from the raw data of dilatometry in the case of a carbon bearing steel.

Lattice parameter is influenced by hydrostatic component of elastic strains. During α transformation on cooling, three kinds of eigen strains ($\varepsilon_{ij}^{*}$) appear as mentioned above. They include (1) transformation strain (about 0.327%), (2) thermal misfit strain (about 0.032%) and (3) C enrichment in γ (0–0.647%). The internal stresses are caused by $\varepsilon_{ij}^{*}$, but high local internal stresses must be relaxed by diffusion and/or dislocation motion at elevated temperatures. The plastic relaxation is too difficult to take into consideration quantitatively because its mechanism is uncertain. However, the unrelaxed state could be evaluated using the Eshelby inclusion theory [74]. The averaged internal stresses, i.e. phase stresses caused by $\varepsilon_{ij}^{*}$ can be written as [75,76]

(9) $$\sigma_{ij}^{\gamma }=-f_{a}C_{ijkl}\left(S_{klmn}-I\right)\varepsilon_{mn}^{*}$$

(10) $$\sigma_{ij}^{a}=\left(1-f_{a}\right)C_{ijkl}\left(S_{klmn}-I\right)\varepsilon_{mn}^{*}$$

where $C_{ijkl}$ and I refer to elastic moduli and unit tensor, respectively. Assuming spherical grain and the Poisson ratio of 0.30, the Eshelby tensor, $S_{ijkl}$, are expressed as $S_{1111}=S_{2222}=S_{3333}=\;\frac{7-5\nu }{15\left(1-\nu \right)}=0.524$, $S_{2233}=S_{3311}\text{\textbackslash{}break}=S_{1122}\;=\;S_{3322}=\;S_{1133}=S_{2211}=\;\frac{5\nu -1}{15\left(1-\nu \right)}=0.0476$, $S_{1212}=S_{2323}\;=\;S_{3131}=\;\frac{-\left(4-5\nu \right)}{15\left(1-\nu \right)}=-0.238$ [77]. Here, three kinds of origins for $\varepsilon_{ij}^{*}$ can be regarded to have isotropic nature, $\varepsilon_{ij}^{*}$ = *q* when i = j and $\varepsilon_{ij}^{*}=0$ when i≠j. Employing the general isotropic Hooke’s equation for $C_{ijkl}$, $\sigma_{11}^{\gamma }=\sigma_{22}^{\gamma }=\sigma_{33}^{\gamma }=-f_{a}\times \left(3.57Eq\right)$ can be obtained, where *E* stands for Young modulus. Needless to say, the sum of phase stresses is zero,

(11) $$\sigma_{ij}^{\gamma }\times f_{\gamma }\;+\sigma_{ij}^{a}\times f_{\alpha }\;=0$$

The elastic internal strain contributing to diffraction peak shift is described by $\beta_{11}=\frac{1-2\nu }{E}\sigma_{11}$, where $\beta_{11}=\beta_{22}=\beta_{33}=\beta$. In γ, tensile hydrostatic strain increases in the beginning of α transformation caused by transformation strain and thermal misfit strain. With increasing of $f_{\alpha }$, it would be expected that the influence of C enrichment ③ becomes larger, which must cancel out the influence of ① and ②. That is, the value of β is postulated to become larger in the beginning and then decrease with progressing of γ→α transformation. In conclusion, the lattice parameter of γ could be expressed by

(12) $$\begin{array}{rl} & a^{\gamma }\left(T,C,\varepsilon \right)=\left(a^{\gamma }\left(RT,0,0\right)+0.0033C\right)\left(1+K^{\gamma }T\right)\\ & \;\;\;\;\;\;\;\;\;\;\;\;\;\;\;\;\;\;\;\;\;\;\;\;\;\;\;\;\;\;\;\;\;\;\;\;\;\left(1+\beta \right) \end{array}$$

**Figure 15.**
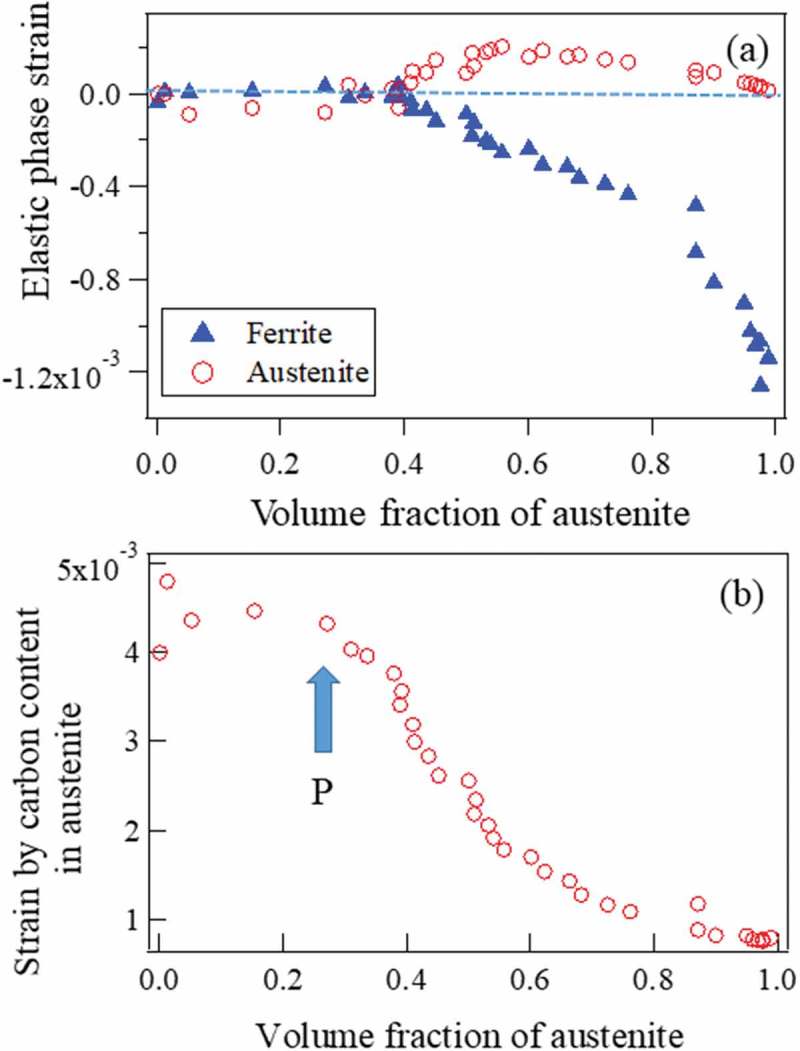
Elastic phase strain (a) and strain caused by C enrichment (b) as a function of volume fraction of austenite. Reproduced with permission from Tomota et al. [7]. Copyright 2018 Iron and Steel Institute of Japan.

The calculated elastic strains in α are plotted in Figure 15. Using the equilibrium stress condition for phase stresses, the elastic strains of γ were computed and the results are also plotted in Figure 15. As was discussed above, at the early stage of γ→α transformation, the stress partitioning is remarkably observed which is mainly caused by ① and ②. The lattice strains caused by C enrichment in γ were evaluated by subtracting the internal elastic strains given in Figure 15(a) from the strains directly determined and the obtained results are presented in Figure 15(b) revealing the behavior of C enrichment. From this figure, the C concentration is found to increase with increasing of $f_{\alpha }$ and cease the increasing after the point P where pearlite transformation starts to take place. As discussed above, the pearlite transformation was considered to occur after the C concentration reached approximately 0.76 mass% where $f_{\gamma }$ was 0.26. The cementite diffraction peaks could not be detected clearly in a 60 s time-sliced diffraction profile because of its small amount, but FWHMs of α peaks were found to increase particularly after the point P, *i.e*. the start of pearlitic transformation. Generally, diffraction line broadening is influenced by the density, character and arrangement of dislocation, coherently diffracting mosaic size and population of planar defects [78]. The present line broadening related to pearlitic transformation is most likely caused by the coherent interface misfit strains [79]. According to high-resolution TEM studies [80], the interface between α and cementite phases in pearlite structure is semi-coherent having a certain crystal orientation relationship, where the interface coherent stresses would be partially relaxed by dislocations and interface ledges.

## 5. Effect of ausforming on martensitic transformation behavior

**Figure 16.**
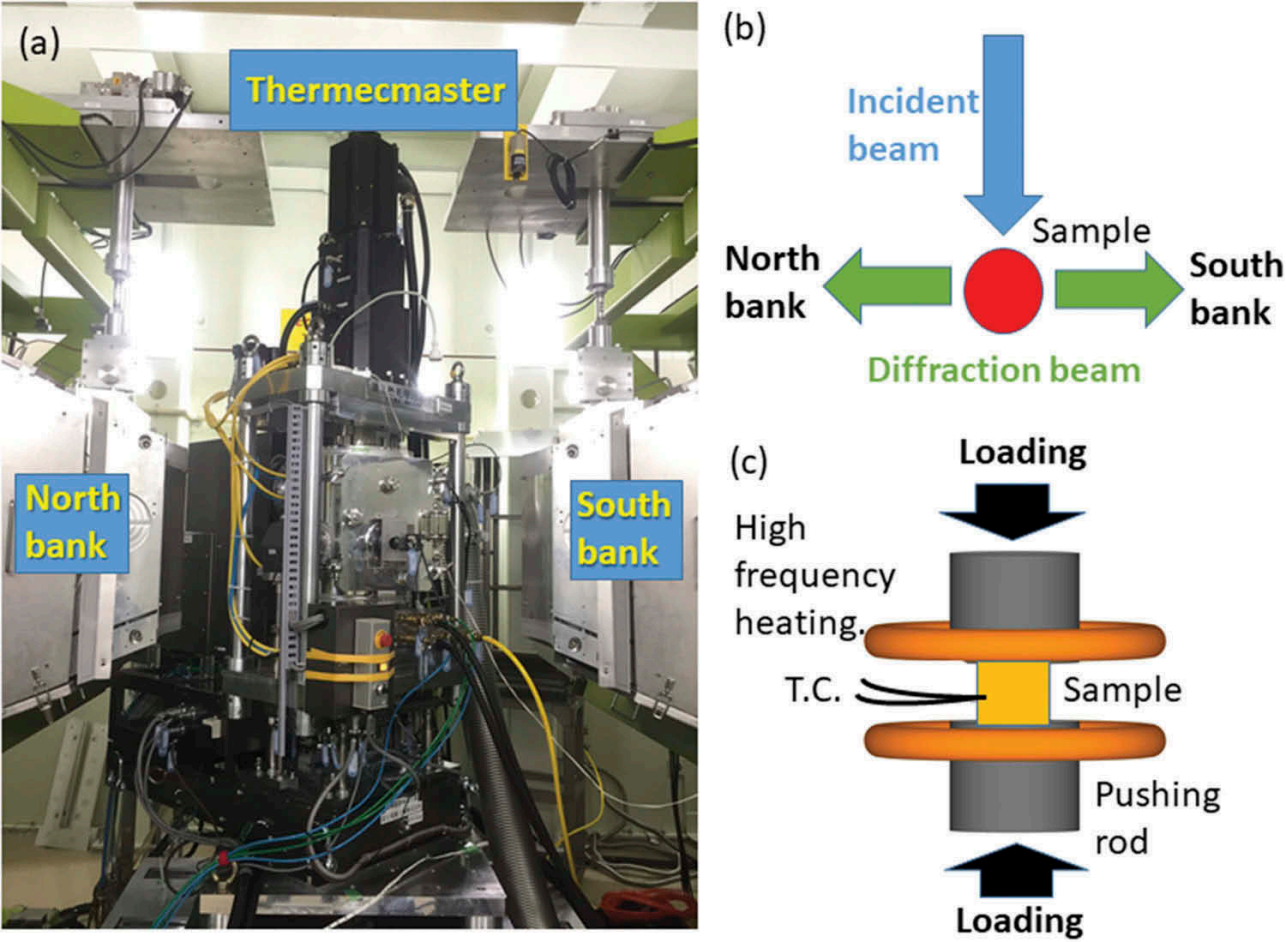
*In situ* neutron diffraction examination during thermo-mechanically controlled processing (TMCP): (a) a TMCP simulator, ‘Thermecmastor-Z’ installed at BL19 of MLF J-PARC [82], (b) top view illustration of a specimen and neutron beam for diffraction measurement and (c) side view. T.C. stands for thermocouple.

Martensitic transformation behavior with or without ausforming was monitored by *in situ* neutron diffraction at BL19, MLF J-PARC using a medium carbon low alloyed steel (0.40C-1.07Cr-0.80Mn-0.21Si-0.21Mo in mass%) [8,9]. A specimen with 6 mm diameter and 11 mm length was austenitized at 1000°C, then cooled down to 700°C to give 40% compressive deformation with a strain rate of 0.4 s^−1^ followed by quenching with He gas flow using a TMCP simulator, ‘Thermecmastor-Z’ installed by Kyoto University [81,82]. Temperature was controlled by a thermocouple welded at the specimen surface with induction heating. The second specimen was directly quenched from 1000°C without ausforming. The experiment view at BL19 is presented in Figure 16 [83], where two detector banks were used for data acquisition with the event mode. Hence, the interval of time slicing can be freely changed after the measurement taking into account the statistic accuracy. Microstructures of these two quenched specimens were found to consist of martensite containing the small amount of retained γ.

**Figure 17.**
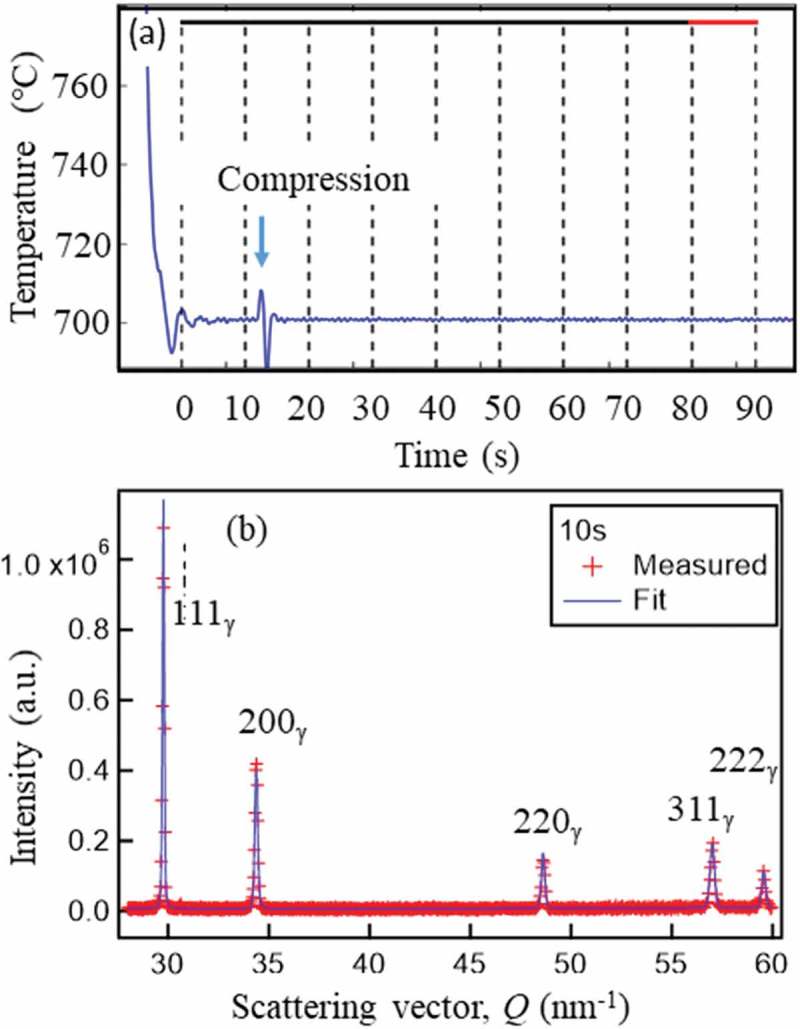
Heating and loading history (a) and diffraction profile obtained in 10 s (from 20 to 30 s in (a)) fitted by the CMWP method. Reproduced with permission of Wang et al. [9]. 2019 Copyright Acta Materialia.

To examine the dislocation density introduced by ausforming at 700°C, the third specimen was austenitized at 1000°C, cooled down to 700°C and then compressed by 40% and kept there for 600 s. The partial temperature history is given in Figure 17(a), in which a little super cooling before holding at 700°C and elevating temperature with plastic compressive deformation were observed. Then, diffraction profiles were taken by slicing interval time of 10 s. The diffraction profile obtained from the first 0–10 s is drawn in Figure 17(b) revealing the specimen was composed of shingle γ phase. The dislocation density was estimated by the CMWP method [55,56] and the example of 200 diffraction peak after compression, i.e. from 20 to 30 s is shown in Figure 17(b). The resultant dislocation density was 1.24 × 10^8^ m^−2^ and 4.40 × 10^14^ m^−2^ m^−2^ before and after compression, respectively. When time interval was increased from 10 to 30 s in Figure 17(a), the dislocation density of 3.17 × 10^14^ was obtained suggesting the progress of recovery. As the results of changing the slicing time, 10 s would be the minimum to achieve statistically reliable fitting. At this ausforming experiment, the operation power of J-PARC was 400 kW which will increase up to the designed 1 MW and therefore the slicing time would be shortened to a few seconds in future. Presently, dislocation density is postulated from flow stress at elevated temperature for TMCP research and development in steel engineering. So, the direct quantitative measurement of dislocation density and arrangement is expected to study dynamic and/or statistic recovery and recrystallization during TMCP, which is very helpful for the scheduling of multi-pass rolling. Hence, to determine the dislocation density during TMCP in a short time at elevated temperature is one of the objectives of the ISMA project.

**Figure 18.**
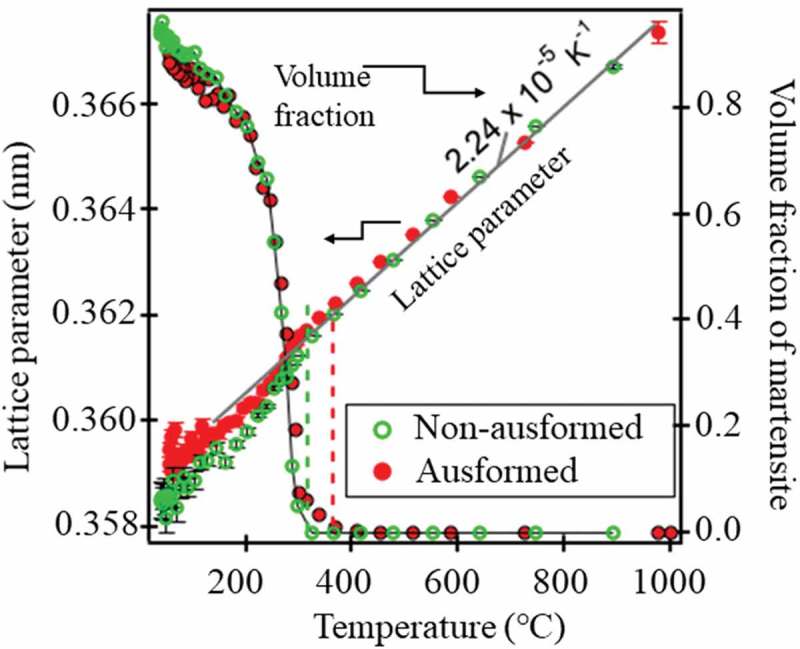
Changes in γ lattice parameter during quenching with or without ausforming for a Cr-Mo-C steel. Reproduced with permission of Wang et al. [9]. 2019 Copyright Acta Materialia.

To determine the diffraction peak positions or *fγ* using the Z-Rietveld software for a whole profile, 2 s sliced data were available with no serious problems. The changes in martensite volume fraction and lattice parameters of γ are plotted in Figure 18. The effects of ausforming include, (1) a little raising martensite staring temperature (Ms), (2) slight decrease of the amount of the retained γ at RT and (3) evolution of transformation texture inherited from γ deformation texture. To be noted in Figure 18 is the temperature dependence of γ lattice parameter; in spite of expansive transformation, it decreased with increasing of martensite volume fraction. Concerning the γ phase stress, the contradicting conclusions have been reported by using laboratory or synchrotron X-ray diffraction so far [83–89] and currently still open for question. This is quite puzzling not to satisfy Equation (11). Synchrotron X-ray diffraction during rapid cooling was applied by Yonemura et al. [38,39] and ex-situ novel EBSD technique was used by Miyamoto et al. [90] but their results were still insufficient to elucidate the internal stress generation upon martensitic transformation. Therefore, a series of neutron diffraction experiments during martensitic transformation has been under investigation at MLF J-PARC for Fe-18Ni [91], Fe-33Ni and Fe-27Ni-0.5C [92], Fe-25Ni-0.4C [93], low alloyed medium carbon steels [9], and Fe-Ni-Co-Ti shape memory alloys [94]. It is interesting that the degree of this deviation from thermal contraction curve becomes smaller by ausforming in Figure 18, suggesting the possible role of lattice defects like dislocations and vacancies introduced with martensitic transformation.

**Figure 19.**
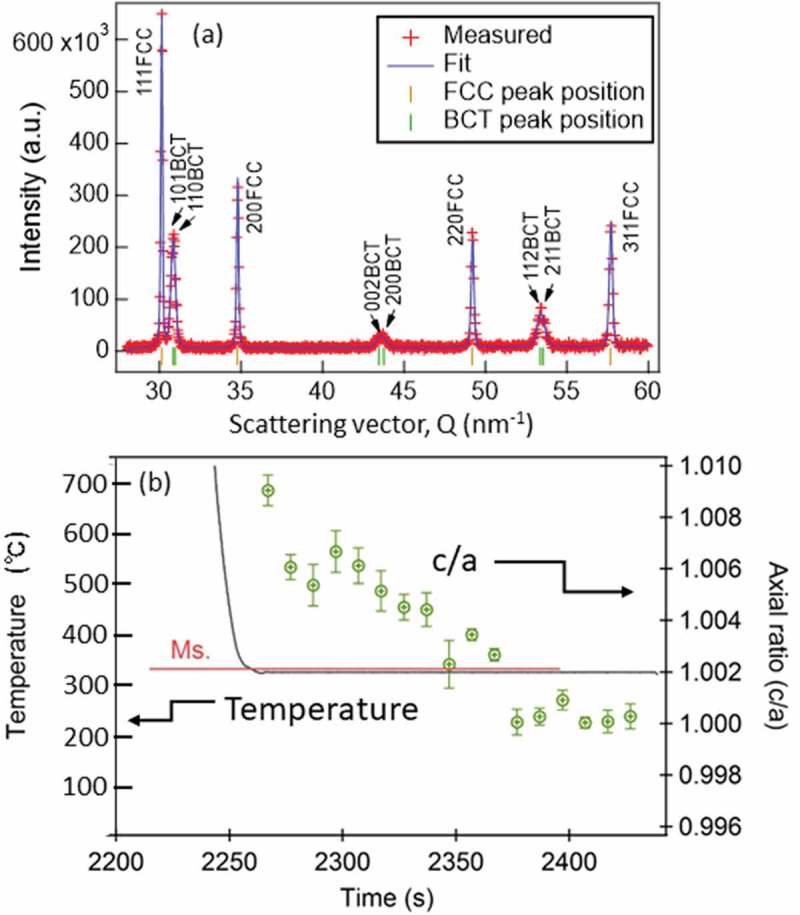
Diffraction profile obtained during quenching which was refined with the Z-Rietveld software (a) and change of the axial ratio c/a of bct martensite with elapsing time (b). Reproduced with permission of Wang et al. [9]. 2019 Copyright Acta Materialia.

Though the martensite studied above was regarded as body-centered cubic (bcc) in Figure 18, more precise fitting assuming body-centered tetragonal (bct) resulted in the change in the axial ratio c/a, which is shown in Figure 19. Concerning another discussing point on the c/a ratio, Honda and Nishiyama proposed the next relationship [95,96]:

(13) $$c/a\;=\;1.000\;+\;0.045\;x\;C\left(mass\%\right)$$

It has been confirmed in various kinds of C bearing steels after their work. For example, Sherby et al. have claimed that the crystal structure of a ferrous alloy with C content less than 0.6 mass% is bcc whereas that with higher than 0.6 mass% C is bct holding Equation (13) [97,98]. Their conclusion has been referred in a textbook [99]. Contrary to their conclusion, Cadeville et al. have shown the c/a of FeCx alloys (≤0.05) martensite prepared by splat quenching from liquid is bct [100]. In addition, the Rietveld analysis has been developed and recently applied to the determination of crystal structure of low or medium C martensite. As results, the understanding has been altered [101–103]; these reports claim that ‘the goodness to fit’ is better assuming bct rather than bcc structure in the Rietveld refinement for X-ray diffraction profile. Among them, Lu et al. have proposed the following relationship instead of Equation (13) for engineering low or medium C bearing steels [103].

(14) $$c/a=c/a=1+0.031C\left(mass\%\right)$$

Quite recently, a novel EBSD analysis was applied to determine a local c/a ratio and revealed that it was different in martensite grain by grain, suggesting that grains yielded earlier showed lower c/a ratio due to auto-tempering on cooling to RT [104,105]. Precise dilatometry has claimed discontinuous progress of lath martensite formation during cooling [106,107]. Referring to these advanced observations, it is interesting to examine fresh martensite just formed near Ms temperature by in situ neutron diffraction. Such an examination was performed [8] and as seen in Figure 19 the c/a ratio of fresh martensite of 0.4 mass% C martensite was higher in the beginning and decreased soon with elapsing time. That is, auto-tempering would be directly monitored. The transformation occurring during isothermal holding below Ms temperature is either bainitic transformation [108,109] or martensite [110]. In the case of engineering steel like the present studied steel, it is extremely difficult to distinguish them from each other.

## 6. Concluding remarks

The progress in global-averaged characterization of microstructure and its evolution during heat treatment or TMCP with neutron diffraction was reviewed using the recent works of the present author’s group [5–9]. Global microstructure data averaged from several million grains are closely related to mechanical properties, which is an attractive merit to use neutron. With increasing of neutron beam intensity, slicing time is becoming shorter but a few second must be needed for the determination of dislocation density by line profile fitting. Attractive progress has also been made using small-angle neutron scattering and transmission Bragg-edge imaging, for which a compact neutron source can be used satisfactorily, so that a new compact neutron source is now under construction at AIST to be used through a network with other compact neutron sources at Hokkaido University [2] and RIKEN [3] in the NEDO/ISMA project [4]. Complementary use of J-PARC [1] and compact neutron sources [2,3] will effectively promote the development of structure materials in Japan. And the combination of neutron experiments with other techniques like EBSD and synchrotron X-ray techniques is becoming more and more important.
