# Supplementary material for: Crystallographic characterization of steel microstructure using neutron diffraction
Source: Sci Technol Adv Mater. 2019 Dec 2;20(1):1189–206. doi: 10.1080/14686996.2019.1699389 (PMC7008240; doi:10.1080/14686996.2019.1699389)
Supplement: Supplemental Material [file TSTA_A_1699389_SM2589.pdf]

CAMP-ISIJ Vol.32 (2019)-334

SCM 鋼マルテンサイト変態のサーメックマスターを用いた中性子回折による検討
(中性子回折法によるマルテンサイト変態の再検討　第 4 報)

Martensitic transformation of SCM steels studied by neutron diffraction with thermecmastor

(Revisit of martensitic transformation using neutron diffraction, Part 4)

物材機構　王 延緒，友田 陽，大村 孝仁，京大　ゴン　ウー，

原子力機構　諸岡　聡，ハルヨ　ステファヌス

1. はじめに

マルテンサイト変態その場直接観察が種々な実験手法で試みられ新たな知見が報告されるようになった。高温でオーステナイトを加工した後に急速冷却する実験には透過能の高い中性子回折の利用が適当で他の手法は使いがたい。本研究では実用鋼を用いてマルテンサイト変態挙動の時分割計測に挑戦した。

2. 実験方法

SCM420 および SCM440 相当の実験室溶解鋼を用いて，J-PARC MLF の BL19 工学材料回折装置・匠に加工熱処理シミュレーター (Thermecmastor)を取り付けて 2 種類（加工の有無）の焼き入れ実験中にその場中性子回折測定を行った。

試料は直径 6 mm，長さ 11 mm の円柱で，1273 K に加熱・保持後に N2 あるいは He ガスにより急冷，あるいは Fig. 1(a)に示すように 973 K まで冷却し 40%の圧縮加工を与えた後に急冷した（加工焼入れ）。圧縮ひずみ速度は 0.4 /s とした。

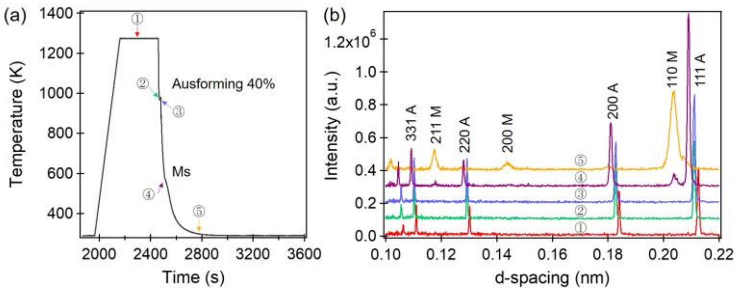

Fig. 1 (a) Thermo-mechanical process for in situ neutron diffraction measurement and (b) diffraction profiles obtained in 4 s at ①1273 K, ②973 K before compression, ③973 K after compression, ④just below Ms and ⑤326 K.

3. 実験結果

Fig. 1(a)のように冷却中にマルテンサイト変態に起因する発熱が見られ，いずれの試料でもマルテンサイト変態が確認された。イベント形式のデータ記録なので、実験後に統計精度を見ながら種々な分割時間（最小 2 s）を採用して冷却・変態中の回折プロファイルを取得した Fig. 1(b)。リートベルト解析の結果によると変態初期のマルテンサイトは SCM420(0.2 mass% C)も SCM440(0.4 mass% C)も BCT（軸比 c/a は 1.05 と 1.09）と判断された。SCM440 を Ms 点直下の温度で恒温保持したところ, 保持時間の経過とともに変態が著しく進行し，回折プロファイルは BCT から BCC に変化し，未変態オーステナイトの格子定数が低下した。マルテンサイト変態の進行に伴いオーステナイトの回折半値幅が大きく増加し，変態後期には約 2 倍となった. 加工焼き入れでは，Ms 点が少し高くなり，オーステナイトに形成された加工集合組織の影響を受けてマルテンサイトにも集合組織が見られた。

本成果は NEDO プロジェクト「新構造材料技術研究開発(ISMA)」の一部である。加工熱処理シミュレーターは ESISM（京都大学構造材料元素戦略研究拠点）のご厚意により借用させていただいた。

Wang Yanxu (National Institute for Materials Science, 1-2-1 Sengen, Tsukuba, Ibaraki 305-0047)
